# Structural Model for Estimating the Influence of Healthy Lifestyle on Episodic Memory in Adults with Subjective Memory Complaints

**DOI:** 10.1155/2020/8349819

**Published:** 2020-03-10

**Authors:** Shijie Li, Yongchuan Tang

**Affiliations:** ^1^Department of Critical Care Medicine, The First Affiliated Hospital of Chongqing Medical University, Chongqing 400016, China; ^2^School of Big Data and Software Engineering, Chongqing University, Chongqing 401331, China

## Abstract

The aim of this study is to examine the relationships between a healthy lifestyle and episodic memory among adults with subjective memory complaints (SMCs). We proposed a structure equation model to study the association between a healthy lifestyle and episodic memory with an investigation covering 309 participants over 50 years old with SMCs. The model showed a good fit after being adjusted (*p* = 0.054, goodness of fit index = 0.981, adjusted goodness of fit index = 0.956, comparative fit index = 0.981, and root mean square error of approximation = 0.049): a healthy lifestyle has a direct positive effect on episodic memory among adults with SMCs (*β* = 0.60). The research model provides possible guidelines for medical staff to prevent the cognitive function decline in the risk population of Alzheimer's disease.

## 1. Introduction

Alzheimer's disease (AD) is a neurodegenerative disorder resulting in progressive memory loss as well as deficits in executive functioning, language, decision-making, and visuospatial abilities [1, 2]. Up to 26 million people are currently affected by AD, costing over 200 billion dollars each year [3]. With the rapid rise in the number of older persons suffering from AD and the associated care burden, there is an increasing focus in maintaining cognitive health in later life. Recently, identifying risk factors and developing primary prevention strategies for promoting cognitive health attract attention widely. Subjective memory complaints (SMCs) refer to subjects who have memory-related complaints without pathological results on neuropsychological tests [4]. SMC is regarded as a factor that may increase the risk of suffering from AD [5]. Jonker et al. reported that over 50% of community-dwelling older adults experience SMC [6], and approximately 6.6% and 2.3% of adults with SMCs will progress to Mild Cognitive Impairment (MCI) and AD, respectively, each year [7]. Neuroimaging studies revealed evidence for brain volume reduction in SMC, resembling those of early AD [8]. Though its etiology is presently unknown, SMCs are viewed as an early indicator of Mild Cognitive Impairment (MCI) or AD.

Episodic memory is conceptualized as the ability to encode, retain, and retrieve knowledge and information about autobiographical events unique to one's experiences. This form of memory involves conscious retrieval of information acquired in a particular place at a particular time [9]. Numerous prospective and retrospective studies show that performance on episodic memory tests is the strongest predictor on which patients will develop AD within the next 1 to 7 years from the initial evaluation [10]. Bäckman and Small find that a general impairment of episodic memory occurs in the early development of AD [11]. Histopathological evidence shows that some of the earliest brain changes in AD occur in the hippocampus and related structures. These regions have been strongly implicated in episodic memory in both lesion and brain imaging studies [12].

People who show increasing SMCs over time are experiencing steeper decline in episodic memory including information storage and immediate recall [13]. An imaging study finds that adults with SMCs are associated with a smaller hippocampus volume [14]. Hülür et al. [15] follow up 15824 middle-aged and elderly people over 50 years old and find that adults with steeper SMCs showed steeper decline of memory performance over time (*ϕ* = 0.49). In addition, the adults with SMCs who show episodic memory decline have a worse cognitive function and a higher risk of developing AD [16]. However, not all of the adults with SMCs have episodic memory decline. What causes this difference? Epidemiological studies found that adults at risk of dementia who adopt a healthy lifestyle tend to perform better in cognitive function [17]. The MacArthur study of successful aging found that healthy lifestyle habits are associated with brain health [18, 19]. Alzheimer's disease report 2014 points to healthy lifestyle behaviors that may lower the risk for AD, including mental and physical activity, diet, and response to stressful stimuli [20]. Based on these researches, the healthy lifestyle of adults with SMCs may affect their episodic memory positively. However, few studies discussed the influence of lifestyle on episodic memory in adults with SMCs at present. Identifying the structural relationship between a healthy lifestyle and episodic memory among adults with SMCs is important to prevent cognitive decline. Thus, the main objective of the present study was to analyze the impact of a healthy lifestyle on episodic memory in adults with SMCs by developing a structure equation model. Specifically, we hypothesized that a healthy lifestyle has a direct positive effect on episodic memory among adults with SMCs.

The rest of this paper is organized as follows: The methods are introduced in Section 2.  Section 3 presents the result of the study. The discussion on the result to the study is presented in Section 4. Conclusions are given in Section 5.

## 2. Methods

### 2.1. Participants

A cross-sectional study is performed over 9 months in 2017-2018 among a convenience sample of 309 participants with SMCs recruited from Tongjiaqiao Community Health Service Center in Chongqing, China. These participants met the study criteria: (1) age > 50 years, (2) permanent local residents, (3) voluntary participation, and (4) ability to communicate in Chinese. The exclusion criteria were as follows: (1) dementia, (2) the total score of the Mini-Mental State Examination-Chinese version less than the cut-off score adjusted for the education level, (3) being unable to comply with the assessment, (4) a terminal prognosis, and (5) impaired reality (e.g., severe hearing impairment).

### 2.2. Measurement

#### 2.2.1. Social Demographic Characteristics

A structured questionnaire was used to collect demographic and health-related factors, including age, gender, years of education, marital status, monthly income, and chronic disease.

#### 2.2.2. Episodic Memory

The episodic memory of participants was measured by the Auditory Verbal Learning Test (AVLT) which is a five-trial learning procedure for a list of 12 words. For the first three times, the interviewer reads aloud a list of 12 words and the respondents are requested to recall those 12 words as many as they could. We record the number of words recalled correctly. It should be noted that (1) words mentioned by the respondents that were not from the AVLT word list were not counted and (2) words mentioned more than once by the respondents were counted only once in each trial. Subsequently, we perform a nonverbal task for 5 minutes. One recorded word counts 1 point. Then, for the fourth trial, the respondents were asked to recall those 12 words as many as possible. Next, we perform another nonverbal task for 20 minutes. Finally, the respondents were asked to recall the listed 12 words as many as possible again. The total score of the AVLT is 60 points. Cronbach's *α* for the Chinese elderly was 0.99 [21].

#### 2.2.3. Healthy Lifestyle

The Health-Promoting Lifestyle Profile-IIR (HPLP-IIR) is a 40-item scale that assesses six defining characteristics of interpersonal relationship, physical activity, health responsibility, stress management, nutrition, and spiritual growth. All subscales are scored as 1-4 (never-always). The 40 items are then aggregated for a total score. A higher score predicts a healthier lifestyle. Cronbach's *α* value for the six dimensions ranges from 0.63 to 0.81 [22].

#### 2.2.4. Mini-Mental State Examination

The Mini-Mental State Examination- (MMSE-) Chinese version (CMMSE) was used to evaluate the cognitive function of participants to exclude the dementia [23]. The CMMSE assesses orientation, memory, attention, calculation, and language ability, and the total score for each participant is 0 point to 30 points. The evaluation criteria of Shanghai Mental Health Center were adopted to assess the cognitive impairment. The cut-off score adjusted for the education level is shown as follows: the illiteracy group (uneducated) ≤ 17 points, the primary school group (education period ≤ 6 years) ≤ 20 points, and the middle school or above (education period > 6 years) ≤ 24 points.

### 2.3. Data Processing

#### 2.3.1. Data Collection

The study was approved by the Tongjiaqiao Community Health Service Center where participants were enrolled. Investigators were trained to administer the CMMSE and AVLT. After meeting the participants, the investigators described the study, obtained informed content, and collected information of participants based on the aforementioned questionnaire one on one.

#### 2.3.2. Data Analysis

Data were double-entered in the EpiData 3.1 software, and multiple logic checks were used to ensure the accuracy. Sociodemographic data was analyzed with descriptive statistical procedures including the mean and standard deviation. The score of episodic memory and healthy lifestyle was analyzed after computing means and standard deviations. Correlations among the variables were analyzed with Pearson's correlation.

Structure equation modeling was conducted to examine the relationship between a healthy lifestyle and episodic memory as well as to reveal the influence from all dimensions of healthy lifestyle on the episodic memory of the adults with SMCs. Age, gender, and education were included as covariates for all the study variables. Based on recommendations for a multifaceted approach to the assessment of the model fit [24], several criteria were used to evaluate the fit. Goodness of fit of the model was evaluated based on the *p* value, *χ*^2^, goodness of fit index (GFI), adjusted goodness of fit index (AGFI), comparative fit index (CFI), and root mean square error of approximation (RMSEA). Satisfactory goodness of fit was defined as *p* > 0.05, *χ*^2^/df < 2.0, GFI > 0.95, AGFI > 0.90, CFI > 0.97, and RMSEA < 0.05, and acceptable goodness of fit was defined as *p* > 0.05, *χ*^2^/df < 3.0, GFI > 0.90, AGFI > 0.85, CFI > 0.95, and RMSEA < 0.08. The SPSS version 18.0 and Amos version 21.0 are the two software programs used to analyze the data. The level of significance was set up at *p* < 0.05.

## 3. Results

Table 1 reports the demographic characteristics of the 309 adults with SMCs who participated in this study. The age of the participants ranges from 50 to 87 (*M* = 65.11, SD = 6.29), where 61.3% were female, 86.1% got married, and 25.57% were at the education level that is lower than primary school. Among the elderly people, 45.9% had more than one chronic disease including hypertension, diabetes, osteoarthritis, obesity, stroke, and coronary disease.

Table 2 reports the score of episodic memory and healthy lifestyle.  Table 3 shows the correlation analysis. Each subscale of healthy lifestyle had a significant positive and moderate to strong correlation with episodic memory (*r* ranged from 0.339 to 0.513, *p* < 0.01).

**Figure 1:**
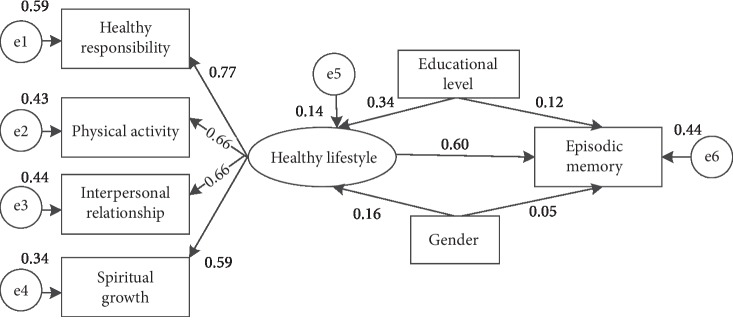
The final reduced model.

The structural equation model was used to evaluate the parameter estimate of and the fit of the proposed relationship in the current study, as shown in Figure 1. The fit indices of the model showed evidence of poor fit in the study model (*p* ≤ 0.001, *χ*^2^/df = 5.016, GFI = 0.897, AGFI = 0.823, CFI = 0.858, and RMSEA = 0.114). We adjust the model by modification indices and parameter estimates to improve the parsimony of the model. Because some paths were insignificant in the hypothesized model, we removed these variables (nutrition, stress management, and age) and tested the modified model. Subsequently, the final model demonstrated a good fit to the data: *p* = 0.054, *χ*^2^/df = 1.729, GFI = 0.981, AGFI = 0.956, CFI = 0.981, and RMSEA = 0.049. As shown in Figure 1, a healthy lifestyle has a direct positive effect on episodic memory among adults with SMCs (*β* = 0.60); four factor-loading parameters (0.59-0.77) in the healthy lifestyle matrix attained significance (*p* < 0.01). The tested model illustrated that individuals with higher health responsibility, more physical activity, better interpersonal relationship, and more spiritual growth can have a better episodic memory.

## 4. Discussion

The study was designed to test the influence of a healthy lifestyle on episodic memory for adults with SMCs. Results demonstrate that a healthy lifestyle has a positive effect on episodic memory of adults with SMCs. A healthy China (2019-2030) program clearly pointed out that the risk population of AD can have a great significance to achieve the goal of decreasing the incidence of Alzheimer's disease by adopting healthy lifestyles including physical activity and reasonable diet and promoting mental well-being [25]. However, having lack of related knowledge and the sense of shame caused by AD lead to the fact that the community-dwelling adults often have a negative attitude towards the prevention and treatment of the disease [26]. Therefore, the medical staff should (1) disseminate the knowledge of AD and its prevention, (2) correct the wrong cognition of residents, (3) focus on the healthy lifestyle level of the risk population, and (4) take intervention to improve sufferers' lifestyle.

The results in Table 3 demonstrated that each subscale of healthy lifestyle had a significant positive and moderate to strong correlation with episodic memory, which is similar with previous studies. Thus, it is crucial for adults with SMCs to develop a healthy lifestyle. According to the attitude behavior model, a patient's intention to behave in a certain way has two major determinants. One is the patient's attitudes towards the behavior, and the other is subjective norm, a social factor, which refers to the perceived social pressure to perform or not to perform the behavior [27]. Thus, the result suggests that to make adults with SMCs pay attention to this disease and strengthen the intention of adopting the healthy lifestyle, medical staff should (1) emphasize the seriousness of Alzheimer's disease, (2) test the cognitive function of the risk population regularly to help them understand the memory state of themselves, and (3) point out the importance of adopting a healthy lifestyle for preventing Alzheimer's disease.

In the proposed model, the result shows that healthy lifestyles including health responsibility, physical activity, interpersonal relationship, and spiritual growth have a direct relationship with the level of episodic memory. Specifically, the findings showed that a high level of health responsibility, physical activity, interpersonal relationship, and spiritual growth is directly linked to a better episodic memory. In line with these findings, a previous research reported that adults with high responsibility about their health are more likely to take healthy behaviors [28] and have a better health condition. Likewise, recent literatures suggested that physical activity can reduce the amyloid deposition of the brain, and doing exercise can reduce dementia risk indirectly by helping keep at bay cardiovascular disease including hypertension, diabetes, and hypercholesterolemia [20]. Furthermore, the findings of this study support the report that good interpersonal relationship is a protect factor for dementia [29]. In Eastern culture, spiritual growth is regarded as striving to higher goals, valuing human life, and constructing the meaning of the life [30]. People who have a good mental condition and goal for a better life are more likely to take measures to maintain health. Therefore, prompting the mental health and setting goals may contribute to adults with SMCs adopting healthy lifestyle and delaying their cognitive decline. It should be noted that the current research is lacking significant associations between nutrition, stress management, and episodic memory, which is inconsistent with previous studies [31, 32]. The difference may be related to the content of the nutrition dimension that does not include all the diet about preventing AD, which may lead to the difference between the results and those of previous studies. Besides, in our investigation, most participants are retired urban residents, which means many of them have a leisure life, so they do not have too much stress. Future studies with different settings and many more participants may provide more insightful knowledge regarding the relationships between managing stress and episodic memory.

In this study, social demographic characteristics including gender and education level are correlated directly with episodic memory. The male and the less educated participants have a worse episodic memory. These findings are in accordance with the research reported by Guo et al. [21], indicating that the AVLT of male is significantly lower than that of female and the score of AVLT was positively correlated with the level of education. An epidemiological study shows that the female has a higher risk of developing MCI or AD than the male [33], but this study has a contrary conclusion. The reason may be that not all memory complaints are related to objective cognitive deficits, while the self-reported memory decline of the female is due to depression rather than objective memory deficits [34]. Prior studies suggested that less education is associated with a higher risk of AD. A low educational level is thought to result in vulnerability to cognitive decline because it results in less-cognitive reserve, which enables people to maintain cognitive function despite brain pathology [35]. It is noteworthy that age, as an important risk factor affecting cognitive function, has not entered into the model. The reason could be that there are many patients with hypertension and diabetes in this study, so age may be a less powerful risk factor for cognitive impairment when these cardiovascular diseases have been taken into account [36].

The study also investigated the association among gender, levels of education, age, and healthy lifestyle. Gender is significantly associated with a healthy lifestyle. Compared to male, the female has a higher healthy lifestyle level, which is in line with the findings in [37]. The findings of this study support the report from earlier studies that a higher level of education may contribute to adopting a healthy lifestyle [37]. The age and healthy lifestyle have no significant association in the current research, which is in contrast to the previous findings where a negative correlation is between age and healthy lifestyle [38]. We argue that the community health center enrolled in the survey may contribute to a higher healthy lifestyle for residents. On the one hand, the family doctor in the community health center supplies tailored services about promoting healthy. On the other hand, the family doctor can supervise these residents adopting a healthy lifestyle.

A couple of potential limitations can be potential in this study. On the one hand, we only use the Health-Promoting Lifestyle Profile-IIR (HPLP-IIR) to measure the healthy lifestyle of participants. Although the HPLP-IIR has been widely applied to research healthy lifestyle, the content in HPLP-IIR cannot cover all lifestyles for preventing AD. In the future, it is necessary to develop a scale to measure the lifestyle in the risk population of AD. On the other hand, only some limited predictors are included in the study; other variables that may influence the relationships between healthy lifestyle and episodic memory should be considered for future researches.

## 5. Conclusions

The study provides evidence that a healthy lifestyle has a positive effect on episodic memory among adults with SMCs. The finding is helpful for planning a possible avenue of preventive measures on cognitive function decline and improving the knowledge of adults with SMCs on the delaying of cognitive function decline. Furthermore, the study contributes to a better understanding of the impact of healthy lifestyle on episodic memory by exploring the roles of interpersonal relationship, physical activity, health responsibility, stress management, nutrition, and spiritual growth in preventing cognitive impairment decline. The intervention mechanism of a healthy lifestyle should be established to protect adults with SMCs from cognitive impairment.

For the following research, it is necessary to develop a scale to measure the lifestyle in the risk population of AD. In addition, some other variables that may influence the relationships between a healthy lifestyle and episodic memory should be taken into consideration for further study.

## Figures and Tables

**Table 1:** Background characteristics of the participants (*N* = 309).

Variables	Conditions	*N*	Percentage
Gender	Male	116	36.80%
	Female	193	61.30%

Marital status	Single/widowed/divorced	42	13.80%
	Married	266	86.10%

Age group (years)	50∼54	10	3.24%
	55∼59	47	15.21%
	60∼64	92	31.39%
	65∼69	86	27.83%
	70∼74	50	16.18%
	75∼79	19	6.15%
	≥80	5	1.62%

Year of education	≤6	79	25.57%
	7∼9	125	40.45%
	10∼12	73	23.62%
	>12	32	10.36%

Number of chronic disease	1	86	27.80%
	2	33	10.70%
	3	17	5.50%
	4	6	1.90%

**Table 2:** The score of episodic memory and healthy lifestyle.

Variables	Range	Mean	Standard deviation
Episodic memory	6∼54	26.47	8.53
Healthy lifestyle	51∼149	91.68	18.32
Health responsibility	11∼44	21.13	5.68
Nutrition	11∼24	19.23	2.85
Physical activity	7∼27	14.21	4.16
Interpersonal relationship	5∼20	13.81	3.91
Stress management	5∼20	11.31	4.06
Spiritual growth	5∼20	11.98	3.78

**Table 3:** The correlation of healthy lifestyle and episodic memory.

Variables	1	2	3	4	5	6	7	8
(1) Episodic memory	1.00							
(2) Health responsibility	0.513^∗∗^	1.00						
(3) Nutrition	0.482^∗∗^	0.460^∗∗^	1.00					
(4) Physical activity	0.429^∗∗^	0.516^∗∗^	0.475^∗∗^	1.00				
(5) Interpersonal relationship	0.426^∗∗^	0.505^∗∗^	0.295^∗∗^	0.398^∗∗^	1.00			
(6) Stress management	0.395^∗∗^	0.536^∗∗^	0.295^∗∗^	0.483^∗∗^	0.708^∗∗^	1.00		
(7) Spiritual growth	0.339^∗∗^	0.421^∗∗^	0.384^∗∗^	0.389^∗∗^	0.441^∗∗^	0.462^∗∗^	1.00	
(8) Healthy lifestyle	0.587^∗∗^	0.814^∗∗^	0.637^∗∗^	0.737^∗∗^	0.752^∗∗^	0.787^∗∗^	0.683^∗∗^	1.00

^∗∗^ *p* < 0.01.

## Data Availability

The data used to support the findings of this study are available from the corresponding author upon request.
